# The Evaluation of Relative Left Ventricular Wall Thickness on Echocardiography for the Diagnosis of ATTR Cardiac Amyloidosis

**DOI:** 10.3390/life16040549

**Published:** 2026-03-26

**Authors:** Shunsuke Kiuchi, Shinji Hisatake, Hidenobu Hashimoto, Yoshiki Murakami, Takanori Ikeda

**Affiliations:** Division of Cardiovascular Medicine, Department of Internal Medicine, Toho University Faculty of Medicine, Tokyo 143-8541, Japanhidenobu.hashimoto@med.toho-u.ac.jp (H.H.); takanori.ikeda@med.toho-u.ac.jp (T.I.)

**Keywords:** echocardiography, relative left ventricular wall thickness, transthyretin cardiac amyloidosis, ^99m^Tc-labeled pyrophosphate scintigraphy

## Abstract

Background: The number of patients with transthyretin amyloid cardiomyopathy (ATTR-CM) has been increasing recently, and the early diagnosis and treatment of it are important. ^99m^Tc pyrophosphate scintigraphy (^99m^Tc-PYP) plays a key role in the early diagnosis of ATTR-CM. In patients who underwent ^99m^Tc-PYP, the early diagnosis of ATTR-CM by echocardiography was evaluated, focusing on left ventricular myocardial form and left ventricular wall thickness. Methods: The present study was conducted on 144 patients who underwent ^99m^Tc-PYP between February 2020 and March 2024. A comparison was made between the ^99m^Tc-PYP positive (P) and negative (N) groups, and significant factors were subjected to multivariate analysis. Results: 17 of 144 patients were positive (14.9%), and 15 patients were diagnosed with ATTR-CM by myocardial or skin (fat) biopsy. Other positive patients were also clinically considered to have ATTR-CM based on findings such as poor cardiac function and cerebral hemorrhage. ^99m^Tc-PYP positive had a significantly larger CTR (60.3% in the P group vs. 53.9% in the N group, *p* = 0.007) and a larger left atrial diameter (42.8 mm in the P group vs. 40.0 mm in the N group, *p* = 0.047). On the other hand, the mean LV wall thickness was significantly thicker (15.7 mm in the P group vs. 12.8 mm in the N group, *p* < 0.001); however the LV end-diastolic diameter was smaller (41.9 mm in the P group vs. 48.4 mm in the P group, *p* < 0.001). The LV mass was similar in both groups, thus the relative left ventricular wall thickness (RWT), which indicates relative wall thickening, was significantly higher in the P group (0.85 in the P group vs. 0.52 mm in the N group, *p* < 0.001). The receiver operating characteristic curve of RWT for assessing ^99m^Tc-PYP positivity had a cut-off value of 0.717 (area under the curve 0.862, 95%CI 0.763–0.961). Conclusions: The evaluation of wall thickness and RWT on echocardiography is important for diagnosing ATTR-CM.

## 1. Introduction

Wild-type transthyretin amyloid cardiomyopathy (ATTR-CM) has a poor prognosis, with a reported 4-year survival rate of approximately 50% [1]. Although no significant improvement was observed over time during this study period, the efficacy of tafamidis for ATTR-CM was reported in 2018 [2], marking a major advance in treatment. Currently, in Japan, the oral tetrameric (TTR) protein stabilizers tafamidis and acoramidis, as well as the injectable RNAi vutrisiran, which inhibits TTR protein production, are approved for the treatment of ATTR-CM. The ATTR-ACT trial reported that tafamidis improved the 30-month survival rate of patients with ATTR-CM from approximately 60% to 70% [3]. However, these medications (disease-modifying medications) for ATTR-CM only slow the progression of the disease, not reverse it. Therefore, the early diagnosis of ATTR-CM and the early administration of disease-modifying medications are necessary. Our hospital also experienced a patient in which it took two years for tafamidis to be administered due to a delay in biopsy, which was essential for the diagnosis at the time [4]. During this time, this patient developed atrial fibrillation and complete atrioventricular block and died early after the administration of tafamidis. This patient may have had a different outcome if earlier diagnosis had enabled the earlier administration of tafamidis. According to the current Japanese statement, biopsy is not necessarily required; however, cardiac accumulation by ^99m^Tc pyrophosphate scintigraphy (^99m^Tc-PYP) is required. Therefore, it is important to appropriately perform ^99m^Tc-PYP in patients with a high possibility of ATTR-CM. We have previously reported on the detection of such patients based on medical history and electrocardiogram changes [5]. In clinical practice, echocardiography is often performed before ^99m^Tc-PYP, following blood examinations, electrocardiograms, and chest X-rays. In the present study, we investigated the usefulness of echocardiographic findings in detecting positive patients of ^99m^Tc-PYP.

## 2. Materials and Methods

The present study was a single-center, retrospective observational study conducted in accordance with the Declaration of Helsinki. The Ethics Committee of Toho University Omori Medical Center approved the present study (approval number: M24196 23100). Information about the present study was posted on the websites of Toho University Omori Medical Center and the Department of Cardiovascular Medicine. An opt-out form was used, and study subjects were given the opportunity to decline the use of their data (a waiver of informed consent from study participants).

### 2.1. Study Participants

The present study was conducted on 144 patients (aged 23–89 years) who underwent ^99m^Tc-PYP between February 2020 and March 2024. ^99m^Tc-PYP was performed on patients who had left ventricular (LV) wall thickness ≥12 mm or were suspected to have ATTR-CM by the attending physician based on clinical findings including the red flag sign [6]. These patients primarily had a history of hospitalization for heart failure (HF) and were candidates for disease-modifying medications for ATTR-CM.

### 2.2. Study Outcomes

A comparison was made between the ^99m^Tc-PYP positive (P) and negative (N) groups. Multivariate analysis was performed for factors that showed significant differences between two groups. The aim of the present study was to identify factors associated with ^99m^Tc-PYP positive patients, with particular focus on echocardiographic findings.

### 2.3. Clinical Findings

The baseline characteristics, medical history/comorbidities, and physical findings of subjects were evaluated using electronic medical records. As the baseline characteristics, we investigated age, gender, height, weight, body mass index and New York Heart Association (NYHA) classification, as well as, the presence or absence of hypertension (HT), diabetes mellitus (DM), and chronic kidney disease (CKD) as medical history and comorbidities. We assessed HT, DM, and CKD through medication history or respective guidelines. The severity of HF was assessed with NYHA classification and brain natriuretic peptide (BNP) on laboratory findings.

### 2.4. Physiological Examinations

We evaluated 12-lead electrocardiogram and transthoracic echocardiographic findings (echocardiography) as physiological examinations. These examinations were assessed by two physicians and technicians who were blinded to the present study. Twelve-lead electrocardiograms were evaluated for the presence or absence of low voltage, which is one of red flag sign [6], and intraventricular conduction disorders. We assessed automatically measured conduction time (PQ interval, QRS interval, QTc interval) and block (the presence or absence of first-degree atrioventricular block (PQ duration of 200 ms or more) and left bundle branch block) as intraventricular conduction disorders. We calculated QTc intervals, which were calculated using the correction formula of the Bazett method [7]. Echocardiography assessed cardiac morphology and function, and LV ejection fraction (EF) was used to assess cardiac systolic function. The proportion of patients with HF preserved EF (HFpEF) (LVEF 50% or higher) [8] was also compared. We calculated LVEF using the modified Simpson method (apical two- or four-chamber view) or the Teichholz method (parasternal long-axis view) [9]. Left atrial (LA) volume (LAV) and LV mass (LVM) were also measured, and indexes were calculated based on height and weight. Relative wall thickening (RWT) was also assessed as a pattern of LV hypertrophy. RWT was calculated with LV posterior wall thickness (LVPWT) × 2/LV diastolic diameter [10]. To evaluate RWT, we used LVPWT included in the Kumamoto criteria, which are useful for the diagnosing ATTR-CM in Japan [11].

### 2.5. Other Examination Findings

In addition, ^99m^Tc-PYP, laboratory data and chest X-rays were also investigated. in ^99m^Tc-PYP, planar and SPECT images were obtained 1 and 3 h after injection of pyrophosphate. Cardiac accumulation was confirmed on SPECT images, and the heart-to-contralateral chest (H/CL) ratios and grade classification were assessed on planar images. From laboratory results, we evaluated C-reactive protein (CRP), electrolytes (sodium and potassium), liver function (total bilirubin (T-Bil), aspartate aminotransferase (AST), alanine aminotransferase (ALT), and lactate dehydrogenase (LDH)), renal function (eGFR, blood urea nitrogen, and creatinine), white blood cell (WBC), hemoglobin, and brain natriuretic peptide (BNP). The eGFR was calculated using the Japanese Society of Nephrology criteria, as follows: eGFR = 194 × Cr − 1.094 × age − 0.287 for men and 194 × Cr − 1.094 × age − 0.287 × 0.739 for women [12]. Moreover, we compared cardiothoracic ratio (CTR) in chest X-rays.

### 2.6. Statistical Analysis

Data were presented as median or percentage. We used the Mann–Whitney U test to compare the groups. In all analyses, differences with *p* < 0.05 were considered statistically significant. Factors that had significant differences in the between-group comparison were evaluated using multivariate analysis. Additionally, the ROC curves for assessing ATTR-CM were created for factor that were significant in the multivariate analysis. EZR (Saitama Medical Center, Jichii Medical University), which is a graphical user interface for R (version 2.13.0, The R Foundation for Statistical Computing, Vienna, Austria) was used for statistical analyses.

## 3. Results

### 3.1. ATTR-CM Diagnosis in the Present Study

Of 144 patients who underwent ^99m^Tc-PYP, 17 patients (11.9%) were positive. The positive rate was approximately 10%, similar to the previous reports from our hospital [5]. ATTR-CM was diagnosed in 15 patients by skin or myocardial biopsy, while the remaining two patients had negative or no biopsy results. To diagnose ATTR-CM using cardiologic nuclear examinations, blood and urine examinations must not detect M protein, and nuclear examinations must confirm cardiac accumulation of Grade 2 or more. Both patients tested negative for M protein, and cardiac accumulation was confirmed on SPECT imaging with ^99m^Tc-PYP. From on the current revised statement, both patients are diagnosed with ATTR-CM. The H/CL ratio measured by ^99m^Tc-PYP was significantly higher in the P group (N group: 1.13, P group: 1.94, *p* < 0.001). Grade classification of the P group also significantly higher than that of N group (N group: 1, P group: 3, *p* < 0.001).

Furthermore, the prevalence of HT was high in approximately 83% of all subjects. There was no significant difference between the two groups; however, blood pressure (BP) was higher in the N group. Patient characteristics are summarized in Table 1. Positive patients were significantly older and smaller, but no gender differences were observed.

### 3.2. Laboratory Examinations and the Severity of Heart Failure

Laboratory data was shown in Table 2. T-Bil, AST, and LDH were significantly higher in the P group. ALT tended to be higher in the P group; however, no significant difference was observed. Renal function was similar between the two groups, and no differences were observed in CRP and WBC, which indicate inflammation. There were also no differences in BNP between the two groups, and no differences in the NHYA classification, which evaluates subjective symptoms. Therefore, it was considered that the severity of HF is roughly equivalent.

### 3.3. Intraventricular Conduction Disorders

Table 3 summarizes electrocardiogram findings. Low voltage, which is a red flag sign, showed no significant differences between the two groups. On the other hand, intraventricular conduction functions, as measured by PQ, QRS, and QTc intervals, were impaired in the P group, with all indices significantly prolonged in the P group. Left bundle branch block is also a common comorbidity [13]; however, no statistical differences were observed between the two groups. First-degree atrioventricular block was significantly more common in the P group, indicating a higher prevalence of intraventricular conduction disorders in the P group, overall.

### 3.4. Cardiac Structural Changes and the Pattern of Left Ventricular Hypertrophy

**Figure 1:**
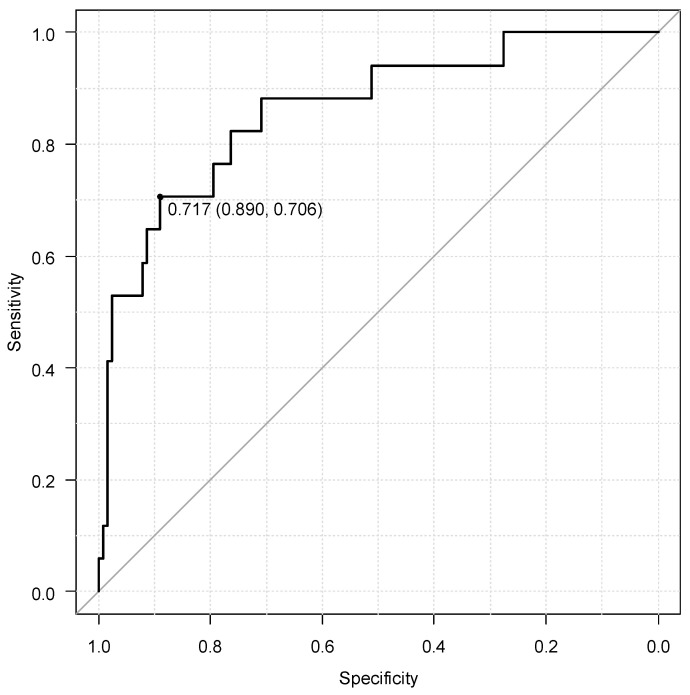
The receiver operating characteristic of relative left ventricular wall thickness for assessing ATTR-CM.

CTR, as shown by chest X-ray, was significantly bigger in the P group. From the echocardiographic results, the LA diameter was significantly longer in the P group; however, there was no difference in the LAV index calculated from body surface area. The LV end-diastolic diameter (LVEDD) was significantly longer in the N group; however, there was no difference in the end-systolic diameter. EF was higher in the N group, on the other hand, there was no statistically significant difference in the percentage of HFpEF. Both the interventricular septum thickness (IVST) and posterior LV wall thickness (PWT) were significantly thicker in the P group (IVST; N group: 13.2 mm, P group: 15.6 mm, *p* = 0.010, PWT; N group: 12.1 mm, P group: 15.6 mm, *p* < 0.001). There was no statistically significant difference in the LVM index (LVMI). RWT, which indicates a cardiac hypertrophy pattern, was significantly higher in the P group. These results are summarized in Table 4. Multivariate analysis was performed focusing on RWT. In all three models, RWT significantly and independently predicted for ^99m^Tc-PYP positive patients (Table 5). The cut-off value of RWT from the receiver operating characteristic curve was 0.717 (area under the curve: 0.862, 95% confidence interval: 0.763–0.961) (Figure 1).

## 4. Discussion

### 4.1. ATTR-CM Diagnosis in Patients Who Underwent ^99m^Tc-PYP

Excluding AL amyloidosis, the diagnostic specificity and positive predictive value of ATTR-CM using ^99m^Tc-PYP has been reported to be 100% [14]. The diagnosis of AL amyloidosis requires the detection of M protein in blood and/or urine; however, M protein was not detected in all subjects in the present study. The subjects in the present study were M-protein-negative and showed cardiac accumulation with ^99m^Tc-PYP, and according to the current statement in Japan, ATTR-CM can be diagnosed. In the present study, ATTR-CM was diagnosed in approximately 10% of patients who underwent ^99m^Tc-PYP, which is not different from previous reports from our hospital [5]. It has also been reported that approximately 15% of Japanese HFpEF patients were ^99m^Tc-PYP positive [15]. From this result, 10% of the ATTR-CM diagnostic rate of the present study was reasonable given the unique nature of this disease, which requires early diagnosis and treatment. On the other hand, in the present study, the H/CL ratio in the ^99m^Tc-PYP positive group was 1.94; however, since the H/CL ratio of >1.6 has been reported to be an independent poor prognostic factor, it may be necessary to consider even earlier diagnosis [16]. Furthermore, because wild-type ATTR-CM is more common in elderly patients, it has been reported that it can take more than a year from onset to diagnosis [17]. The aim of the present study was to identify positive cases in patients who underwent ^99m^Tc-PYP; RWT, which indicates the thickening pattern of the ventricular wall, was useful for detecting them.

### 4.2. Echocardiographic Findings of the ATTR-CM

The Japanese guidelines for cardiac amyloidosis also include information on echocardiography. Two-dimensional method, M-mode method, Doppler method, tissue Doppler method, transesophageal echocardiography, strain Doppler method, and speckle tracking method are each classified as Class I [18]. However, some of these methods cannot be performed at all hospitals. Therefore, an LV wall thickness of 12 mm or more is often used as the initial screening criterion [19]. On the other hand, because diagnosing ATTR-CM based on LV wall thickness alone is difficult, carpal tunnel syndrome and other red flag signs are also used. In echocardiography, in addition to LV wall thickness, the evaluations of LV hypertrophy (LVH) patterns, LV diastolic function, apical sparing and the IWT score were also recommended [20]. This score includes RWT, the ratio between early mitral inflow velocity and mitral annular early diastolic velocity (E/e’), LV longitudinal strain (LS), septal systolic apex-to-base ratio (SAB), and tricuspid annular plane systolic excursion (TAPSE). Among these, RWT was given a higher score. In the present study, RWT was also found to be independently useful for diagnosis, however in this retrospective study, other parameters of IWT score were not assessed in all patients. Therefore, we think it would be useful to combine LV wall thickening with RWT—which is one of the methods used to evaluate LVH—to provide a good indicator that is easily applicable in everyday clinical practice.

In the present study, LVEDD was significantly lower in the P group. Because RWT is calculated from LVEDD and LV wall thickness, it is possible that it may be appropriate to evaluate LVEDD and LVH as with RWT and LVH. In ATTR-CM, LVEDD increases as the disease progresses from transition to HFpEF to HFrEF [21]. In the subjects of the present study, who were diagnosed with an early stage of ATTR-CM, the LVEDD in the P group was prior to enlargement. On the other hand, because LV wall thickness in hypertensive patients is also affected by the number of years they have had HT [22], it is considered that patients with LVH of 12 mm or more, such as those in the present study, had a long history of HT. When high systolic BP due to a long history of HT is accumulated, the higher the cumulative systolic BP, the more advanced the structural changes in the heart, such as LVEDD enlargement [23]. Therefore, differences in RWT, LVEDD, and LV wall thickness occurred in the present study, which captured the early stages of ATTR-CM. Even when evaluation is performed after the disease has progressed, the LV wall progressively hypertrophies in ATTR-CM, and the wall thickness is thicker than in hypertension. In diagnosing ATTR-CM, unlike LVEDD, the diagnostic ability of RWT is maintained regardless of the stage of the disease, and therefore RWT in the IWT score is thought to be high. Other indicators included in the IWT score, such as E/e’, LS, SAB, and TAPSE, were not evaluated in all patients in the present study. These examinations are often difficult for practicing physicians to perform. Therefore, focusing on RWT as a first step in suspecting ATTR-CM from echocardiography is beneficial.

Furthermore, while the cut-off value for RWT in the IWT score is 0.8, the cut-off value in the present study was 0.717. The normal value for RWT is often considered to be below 0.42, and it is important to be aware of cut-off values of 0.7 to 0.8 when proceeding with various examinations such as ^99m^Tc-PYP from both studies.

### 4.3. Left Ventricular Hypertrophy Pattern and ATTR-CM

In the VALLANT sub-study, LV hypertrophy (LVH) was classified into three patterns based on LVMI and RWT [24]. These three patterns are concentric remodeling (normal LVMI and increased RWT), eccentric LVH (eLVH) (increased LVMI and normal RWT), and concentric LVH (cLVH) (increased both LVMI and RWT). A normal pattern without LVH is when the LVMI does not increase and RWT is normal. The present study demonstrated the progression of concentric remodeling in the PYP-positive group. In the present study, LVMI tended to be lower in the P group; however, this result may be due to the small number of subjects. ATTR-CM is often associated with cLVH [25]; however, a similar patient of ATTR-CM with concentric remodeling has been reported [26], and concentric remodeling has also been shown to be useful for diagnosing ATTR-CM in patients hospitalized for decompensated HF [27]. Based on these results, ATTR-CM exhibits an LVH pattern with increased RWT. On the other hand, HT, one of the most common causes of LVH, often exhibits patterns of increased LVMI (eLVH and cLVH), indicating the pattern of LVH is different [28]. In ATTR-CM, a TTR protein produced in the liver dissociates into monomers, forming amyloid fibrils and accumulating in the heart [29,30]. In conditions with LV afterload, such as HT and aortic valve stenosis, cardiomyocyte hypertrophy occurs. These differences in pathology are thought to be reflected in the different patterns of LVH. Similarly, in Fabry disease, which exhibits accumulation cardiomyopathy, cLVH is the most common, with concentric remodeling and eLVH both occurring in approximately 20% of patients [31]. Therefore, focusing on RWT may be useful in patients of cardiomyopathy characterized by LVH. Although strain echocardiography was not performed in the present study, previous studies have shown that apical strain gradients are useful for distinguishing cardiac amyloidosis on cLVH [32].

### 4.4. Study Limitations

The main limitation of the present study is a small sample size, which is a single-center retrospective study. There were 144 patients in total, with only 17 in the P group. Since the P group consisted of only 17 patients, the multivariate analysis had to be divided into three models. This also meant that we could not include a large number of factors. Therefore, the possibility of overestimation cannot be ruled out. Additionally, LVH was assessed only by echocardiography, and imaging examinations such as MRI and CT were not included. These examinations have also been reported to be useful in diagnosing ATTR-CM [33]. Although strain echocardiography and other methods have been shown to be useful in differentiating cLVH from ATTR-CM, this retrospective study did not evaluate these methods during echocardiography, which is another limitation of the present study. To address these limitations, prospective studies involving a larger number of patients are needed.

## 5. Conclusions

In patients with LVH, focusing on RWT on echocardiography may aid in the diagnosis of ATTR-CM.

## Figures and Tables

**Table 1:** Patient characteristics and ^99m^Tc-PYP scintigraphy between groups.

	Negative Group (n = 127)	Positive Group (n = 17)	*p* Value
Age (years)	65	81	<0.001
Male (n, %)	94, 74	11, 65	0.534
Height (cm)	165	154	0.007
Weight (kg)	62.5	53.8	0.060
Body mass index (kg/m^2^)	23.6	21.9	0.329
NYHA class (I/II/III)	6/104/17	0/16/1	0.838
Systolic blood pressure (mmHg)	132	119	0.039
Diastolic blood pressure (mmHg)	78	70	0.083
Pulse rate (bpm)	79	75.5	0.099
Medical history of hypertension (n, %)	105, 83	15, 88	0.710
Medical history of diabetes (n, %)	40, 32	4, 24	0.594
Medical history of dyslipidemia (n, %)	42, 33	8, 47	0.350
Medical history of CKD (n, %)	46, 36	6, 35	0.951
Heart to contralateral ratio of ^99m^Tc-PYP scintigraphy	1.13	1.94	<0.001
Grade classification in ^99m^Tc-PYP scintigraphy (I/II/III/IV)	118/8/1/0	0/2/8/7	<0.001

NYHA: New York Heart Association, CKD: chronic kidney disease. Continuous data are expressed as the median. *p*-values were determined using the Mann–Whitney U test.

**Table 2:** Laboratory findings between the groups.

	Negative Group (n = 127)	Positive Group (n = 17)	*p* Value
Sodium (mg/dL)	140	140	0.237
Potassium (mg/dL)	4.1	4.1	0.617
CRP (mg/dL)	0.1	0.2	0.300
Total-bilirubin (mg/dL)	0.6	1.1	0.049
AST (IU/L)	21	26	0.005
ALT (IU/L)	16	20	0.350
LDH (IU/L)	224	258	0.010
BUN (mg/dL)	19.0	24.0	0.199
Creatinine (mg/dL)	1.08	1.10	0.716
eGFR (mL/min/1.73 m^2^)	51.0	45.9	0.708
White blood cell (/μL)	6300	6200	0.570
Hemoglobin (g/dL)	12.8	13.6	0.445
Brain natriuretic peptide (pg/mL)	218.7	365.0	0.205

CRP: C-Reactive Protein, AST: aspartate aminotransferase, ALT: alanine aminotransferase, LDH: lactate dehydrogenase, BUN: blood urea nitrogen, eGFR: estimated glomerular filtration rate. Continuous data are expressed as the median. *p*-values were determined using the Mann–Whitney U test.

**Table 3:** ATTR-CM has intraventricular conduction defects.

	Negative Group (n = 127)	Positive Group (n = 17)	*p* Value
Heart rate (bpm)	79	75.5	0.099
Atrial fibrillation/flutter (n, %)	13, 10.2	2, 11.8	0.187
Low voltage (n, %)	3, 2.4	1, 5.9	0.107
First-degree atrioventricular block (n, %)	22, 17.3	6, 35.3	0.179
Left bundle branch block (n, %)	9, 7.1	2, 11.8	0.066
PQ duration (ms)	174	200	0.002
QRS duration (ms)	100	131	0.017
QTc duration (ms)	422	473.5	<0.001

Continuous data are expressed as the median. *p*-values were determined using the Mann–Whitney U test.

**Table 4:** Heart morphology in both groups.

	Negative Group (n = 127)	Positive Group (n = 17)	*p* Value
Cardiothoracic ratio in chest X-ray (%)	53.9	60.3	0.007
Left atrial diameter (mm)	40.0	42.8	0.047
Left atrial volume index (mL/m^2^)	35.5	44.0	0.522
Left ventricular end-diastolic diameter (mm)	48.4	41.9	0.001
Left ventricular end-systolic diameter (mm)	30.3	30.8	0.998
Interventricular septal thickness at end-diastole (mm)	13.2	15.6	0.010
Posterior wall thickness at end diastole (mm)	12.1	15.6	<0.001
Left ventricular mass index (g/m^2^)	94.0	65.0	0.792
Relative left ventricular wall thickness	0.52	0.85	<0.001
Left ventricular ejection fraction (%)	65.1	52.0	0.003
The percentage of HFpEF (n, %)	101, 80	9, 53	0.076

HFpEF: Heart failure preserved ejection fraction. Continuous data are expressed as the median. *p*-values were determined using the Mann–Whitney U test.

**Table 5:** Multivariate analysis for assessing ATTR-CM.

	Model 1		Model 2		Model 3	
	OR	*p* Value	OR	*p* Value	OR	*p* Value
Relative left ventricular wall thickness * 10	2.04	<0.001	1.71	0.021	1.84	0.002
Systolic blood pressure	0.97	0.173				
Cardiothoracic ratio	1.12	0.067				
QRS duration	1.03	0.019				
Left ventricular end-diastolic diameter			0.94	0.320		
Left ventricular ejection fraction			0.93	0.014		
Total-bilirubin					2.75	0.438
Aspartate aminotransferase					0.98	0.183
Lactate dehydrogenase					1.01	0.080

The multivariate analysis was performed by logistic regression analysis. * means multiplied.

## Data Availability

The datasets used and/or analyzed during the current study available from the corresponding author on reasonable request.
